# Popular Online Content as a Treatment-as-Usual Control in Digital Mental Health Intervention Trials: Secondary Analysis of Two Online Randomized Controlled Trials With Repeated Measures

**DOI:** 10.2196/83707

**Published:** 2026-04-20

**Authors:** Benjamin T Kaveladze, Stephen M Schueller, David C Mohr

**Affiliations:** 1 Center for Technology and Behavioral Health, Dartmouth College, Hanover, NH, United States; 2 Department of Psychology, School of Social Ecology, University of California, Irvine, 4201 Social & Behavioral Sciences Gateway, Irvine, CA, 92697-7085, United States, 1 (949) 824-5574; 3 Department of Preventive Medicine, Center for Behavioral Intervention Technologies, Northwestern University, Chicago, IL, United States

**Keywords:** treatment-as-usual, control conditions, randomized controlled trials, popular online content, digital mental health

## Abstract

**Background:**

Treatment-as-usual (TAU) conditions are intended to reflect the support typically received in routine treatment settings. For digital mental health interventions (DMHIs) delivered online, TAU conditions should reflect the usual patterns of online help-seeking. The lack of ecologically valid TAU control conditions has been a gap in effectiveness trials of online DMHIs. In this study, mental health–related popular online content (eg, advice TikToks, lived experience vlogs, and self-care infographics) was examined as a valuable TAU control condition.

**Objective:**

This study examined the feasibility of popular online content as a TAU control condition in DMHI trials.

**Methods:**

This study was a secondary analysis of two randomized controlled trials. Both trials recruited participants online, primarily via an online study recruitment platform. In study 1 (N=916), US adults with elevated depression or anxiety were randomized to either (1) complete a single-session DHMI for depression and anxiety (n=291), (2) search the web for popular online content relevant to their struggles (n=312), or (3) search a curated library of mental health–related popular online content (n=313). In study 2 (N=431), US adults with elevated loneliness were randomized to (1) complete a single-session DHMI for loneliness (n=136), (2) search a curated library of popular online content related to loneliness (n=145), or (3) complete an attention-matched control condition (n=150). All 6 programs took approximately 10 to 20 minutes to complete and were entirely self-guided. Participants rated each program’s credibility and expected benefit, as well as their feelings of distress (study 1) and loneliness (study 2). The studies did not involve interaction between participants and the research team.

**Results:**

In study 1, dropout during the treatment was 4.8% (14/291) for the single-session intervention, 25.9% (81/312) for online help-seeking, and 9.6% (30/313) for the curated library. The curated library’s credibility and expected benefit score did not differ from that of the single-session intervention (Cohen *d*=0.08; *P*=.88) and was higher than that of unguided help-seeking (Cohen *d*=0.23; *P*=.01). In study 2, dropout was higher in the curated library condition (7/145, 4.8%) than in the single-session intervention and the attention-matched control condition (0/136, 0.0% and 0/150, 0.0%). The mean credibility and expected benefit score for the curated library was comparable to that of the attention-matched control condition (Cohen *d*=0.00; *P*>.99) but lower than that of the single-session intervention (Cohen *d*=0.32; *P*=.02). Changes in distress and loneliness from baseline to 8-week follow-up did not differ across the conditions in study 1. All effect sizes were small in study 1 (Cohen *d*<0.15*),* and no comparisons were statistically significant *(P*>.06). Similarly, in study 2, all effect sizes were small (Cohen *d*<0.12), and no comparisons were statistically significant (*P*>.25).

**Conclusions:**

Curated libraries of popular online content are a feasible, ecologically valid TAU benchmark for effectiveness trials of online DMHIs. Future research on TAU conditions in online help-seeking contexts should better align with observed DMHI attrition rates and account for the increasingly central role of conversational artificial intelligence in online mental health support.

## Introduction

Research on digital mental health interventions (DMHIs) is evolving from a narrow focus on efficacy toward a broader emphasis on real-world effectiveness. To evaluate the effectiveness of DMHIs relative to leading alternatives, randomized controlled trials (RCTs) require credible treatment-as-usual (TAU) controls that reflect the support participants would likely access in the absence of a DMHI [1]. For DMHIs delivered online, TAU controls should reflect resources typically used by online help-seekers (ie, online help-seeking as usual) [2].

### Popular Online Content as Mental Health Support

Trials of DMHIs have used a variety of controls, including minimal comparators such as waitlists and brief psychoeducation, as well as more active alternatives, such as popular DMHIs or human-supported psychotherapy [2]. However, for DMHIs intended for online delivery, these controls do not constitute TAU because they fail to reflect the resources that are typically accessed by online help-seekers [3]. Instead, online help-seekers are more likely to engage with mental health–related popular online content. Such content—defined as online media intended for a public audience—is ubiquitous, often tailored to specific groups, and, in some cases, may offer valuable guidance and emotional support. As such, it may serve as the foundation for more ecologically valid TAU (ie, online help-seeking as usual) controls in DMHI trials.

Some popular online content is created with the explicit aim of supporting consumers’ mental health (eg, TikToks offering self-care advice, attention-deficit/hyperactivity disorder infographics, and blog posts about a writer’s experience with anxiety). Other content is not explicitly focused on mental health but may nevertheless support relevant goals. For example, a philosophy podcast might promote a positive mindset shift, while a favorite music video could help to regulate difficult emotions. However, few content creators are trained mental health professionals, and all operate within an internet ecosystem that rewards engagement rather than user well-being. Consequently, much mental health–related popular online content is not evidence-based, and some may be unhelpful or even harmful [4]. At the same time, its widespread popularity and use suggests that many individuals find it helpful.

Popular online content has been evaluated in several RCTs. One trial sent participants weekly emails with psychoeducational online content related to positive psychology, including videos and blog posts [5]. Another study repurposed popular videos, articles, and infographics as stress management “microinterventions” to help participants develop self-care skills and reduce stress and depression [6]. Together, these studies provide preliminary evidence that popular online content can be feasible and acceptable in RCT contexts. However, further research is needed to determine how popular online content–based controls compare with conventional controls, and which types of popular content best fulfill the functions of a control (ie, accounting for alternative explanations, such as the natural course of the treatment target, expectations of benefit, and engagement) [1-3].

## Methods

### This Study

In secondary analyses of 2 RCTs [7], this study examined the feasibility of popular online content as a TAU control condition by evaluating the acceptability and efficacy of popular online content–based programs relative to evidence-based DMHIs and a conventional attention-matched control condition.

### Participants

In study 1, participants were recruited globally through advertisements on social media and within the United States via the online participant recruitment platform CloudResearch Connect [8]. To be eligible, participants were required to be fluent in English, be aged 16 years or older, and score at least 3 (range 0‐6) on either the depression or anxiety subscale of the 4-item version of the Patient Health Questionnaire, as these scores are suggestive of an elevated depressive or anxiety disorder [9]. In study 2, US participants experiencing elevated loneliness were recruited via CloudResearch Connect. Eligibility criteria for study 2 included fluency in English, being aged 18 years or older, and meeting predefined criteria for “struggling with loneliness,” defined as distress related to loneliness (binary item) and scoring 6 or greater on the 3-item version of the UCLA Loneliness Scale, reflecting elevated loneliness [10]. In both study 1 and study 2, computer and internet literacy was an implicit eligibility criterion.

### Changes to Methods After Trial Commencement

CloudResearch Connect was not originally planned as a recruitment platform; however, it was used after other recruitment methods were flooded by fraudulent participants (ie, automated bots or individuals falsely claiming to meet eligibility criteria). At the time of this study, CloudResearch Connect appeared to have a low rate of fraudulent research participation.

### Study Procedures

#### Overview

In both studies, aside from recruitment and compensation, all study procedures were conducted via the online survey platform Qualtrics (Qualtrics, LLC), which participants accessed using their personal computers, tablets, or smartphones. Randomization was implemented through Qualtrics’ built-in randomization feature. There was no interaction between participants and researchers during the study, except in a few instances when participants contacted the research team to ask a question or report issues related to the recruitment platform. Researchers were unaware of participants’ condition assignments until data collection was complete. Efforts were made to prevent participants from knowing whether they received an experimental or control intervention; however, blinding was not formally assessed. Studies 1 and 2 were prospectively registered (NCT05687162) on November 29, 2022. OSF preregistration was completed prior to viewing any participant data on April 7, 2023, and July 3, 2023, respectively, for studies 1 and 2. Study 1’s first participant was enrolled on April 5, 2023, and study 2’s first participant was enrolled on July 3, 2023.

#### Study 1

**Figure 1.**
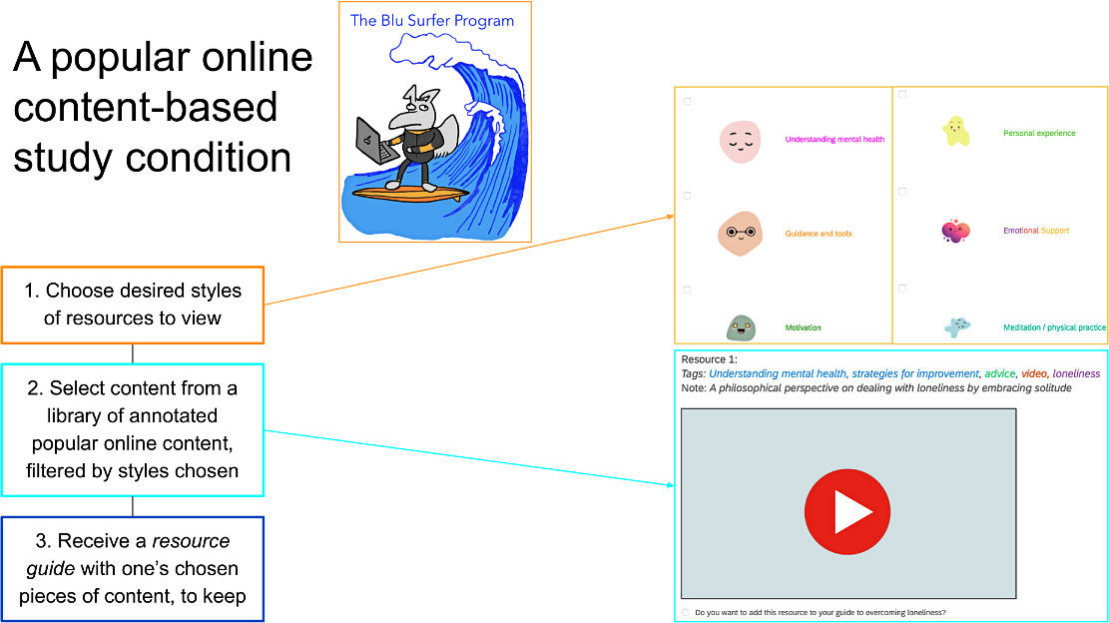
A popular online content-based program for overcoming psychological distress. SSI: single-session intervention.

Participants were randomized in parallel (1:1:1) to one of three 10‐ to 20-minute conditions. The first condition was the Action Brings Change Project, an evidence-based digital single-session intervention for depression and anxiety rooted in behavioral activation [11]. The second condition was unguided online help-seeking, in which participants were instructed to search the web for popular online content to address their mental health concerns and create a personal resource guide of links to the content they found most helpful for future reference. The third condition was a curated popular content library, in which participants indicated the types of supportive resources they wished to view (eg, guidance and tools, motivational content, emotional support) and then browsed a library of up to 120 annotated pieces of popular online content filtered according to their preferences. Participants selected items to add to their resource guide for later viewing (see Figure 1 for a description of the intervention components).

The curated popular online content library aimed to ensure a diverse range of participants could find resources they resonated with. Half of the 120 pieces of content included in the library were crowdsourced from online mental health support communities and undergraduate students using the prompt, “If you could link to one video, image, article, or other online content to help someone struggling with their mental health, what would you send?” The remaining 60 pieces of content were identified by members of the research team through web searches, aiming to include content that was likely to be beneficial for mental health and diverse in format. The library included various kinds of content (eg, 48 videos, 22 articles, 13 infographics, 9 activities or worksheets, and 6 podcasts; the remaining 32 pieces of content were in categories with less than 5 pieces per category).

While unguided online help-seeking was intended to serve as the TAU for the online help-seeking context, the curated version may be conceptualized as an enhanced TAU [12]. Enhanced TAU involves modifications designed to remove variability that could otherwise interfere with or confound trial outcomes. For example, in primary care trials, participating physicians are often trained uniformly to reduce variability related to physician knowledge. Similarly, the curated enhanced TAU in this study isolates the comparison to the source of intervention content (popular online content vs researcher-developed content).

Four and 8 weeks after completing the initial study session (during which participants reported baseline measures, completed their assigned program, then reported postintervention measures), participants were invited to complete follow-up surveys to assess changes in mental health outcomes. The initial session took most participants fewer than 30 minutes to complete, and the follow-up survey took around 5 minutes to complete. Participants were permitted to complete the 8-week follow-up assessment regardless of whether they had completed the 4-week assessment. The study 1 interventions can be accessed through the link provided in Kaveladze [13].

#### Study 2

Participants were randomized in parallel using simple randomization (1:1:1) to one of three 10‐ to 20-minute interventions: (1) a digital single-session intervention for loneliness [14]; (2) a curated popular content library featuring the most frequently selected pieces from study 1, along with additional content focused on loneliness; or (3) an attention-matched control that was structurally similar to the digital single-session intervention but encouraged participants to share their feelings with close others rather than delivering an evidence-based intervention (the program is called The Sharing Feelings Project) [10]. The popular online content conditions in studies 1 and 2 were conceived as active comparators rather than controls; the initial hypothesis was that they would be more efficacious than the single-session interventions [7]. Accordingly, the present examination of popular online content as an online TAU is secondary to the studies’ original aims.

Four and 8 weeks after completing the initial study session (during which participants reported baseline measures, completed their assigned program, then reported postintervention measures), participants were invited to complete follow-up surveys to assess change in mental health outcomes. The initial session took most participants fewer than 30 minutes to complete, and the follow-up survey took around 5 minutes to complete. Participants were permitted to complete the 8-week follow-up assessment regardless of whether they had completed the 4-week assessment. The study 2 interventions can be accessed through the link provided in Kaveladze [15]*.*

### Ethical Considerations

Study procedures were approved by the University of California, Irvine Human Subjects Review Board (protocol 1253) and conducted in accordance with the World Medical Association Declaration of Helsinki. Participants provided informed consent via an online information sheet after eligibility screening. Participation was anonymous, and all procedures were conducted online without direct contact between participants and the research team. The data collection platform (Qualtrics) securely stored participant information. In both studies, participants received US $10.00 for completing the screener, intervention, 4-week follow-up, and 8-week follow-up.

### Measures

Outcomes were assessed at baseline, postintervention, 4 weeks, and 8 weeks, consistent with typical assessment timelines in DMHI trials. All measures were self-reported. In study 1, the primary outcome was psychological distress, assessed using the 9-item Depression Anxiety Stress Scale [16]. The scale asks participants to rate statements about their past week (eg, “I found it hard to wind down”) on a scale from 0 (did not apply to me at all) to 3 (applied to me very much or most of the time), with total scores ranging from 0 to 27. In study 2, the primary outcome was loneliness, measured by version 3 of the UCLA Loneliness Scale [17]. The scale assesses the frequency of feelings such as “left out” or “close to people” on a scale from 1 (never) to 4 (often), with total scores ranging from 20 to 80. In both studies, the Credibility/Expectancy Questionnaire (CEQ) total score (range 4‐67) was used to assess participants’ perceptions of program credibility and expected improvements in the primary outcome [18]. Some CEQ items are rated 1 (not at all) to 9 (very much), and others are rated from 0 (0%) to 10 (100%).

All 3 measures demonstrated reliability and validity in adult samples [16-18]. Measures were presented in a random order at each time point for each participant, and all measures have been used extensively in online questionnaires. Dropout was defined as discontinuing participation in the study while completing the program to which a participant was assigned. Additional outcomes are described in the study preregistrations.

### Statistical Analysis Plan

Analysis of variance was used to compare outcomes across conditions at a single time point, followed by Tukey tests for pairwise comparisons. Linear mixed-effects regression models were used to assess differences across conditions in change over time*.* Chi-square tests and odds ratios (ORs) were used to compare program dropout rates. The primary preregistered analyses in each study evaluated efficacy, whereas secondary preregistered analyses examined feasibility and acceptability. Analyses included all available data under a missing-at-random assumption using an intent-to-treat approach. Statistical significance was defined as a *P* value of .05 or less.

### Sample Size

In study 1, based on effect sizes reported in studies of similar single-session interventions, the aim was to detect a Cohen *d* of 0.20 between conditions. Power analyses indicated that 394 participants per condition would be required to detect a Cohen *d* of 0.20 with 80% power; however, this estimate did not account for attrition. The same target effect size was specified for study 2, but due to an error in the sample size calculation, the study was subsequently underpowered. No interim analyses were conducted, and no stopping guidelines were established.

## Results

### Overview

**Figure 2.**
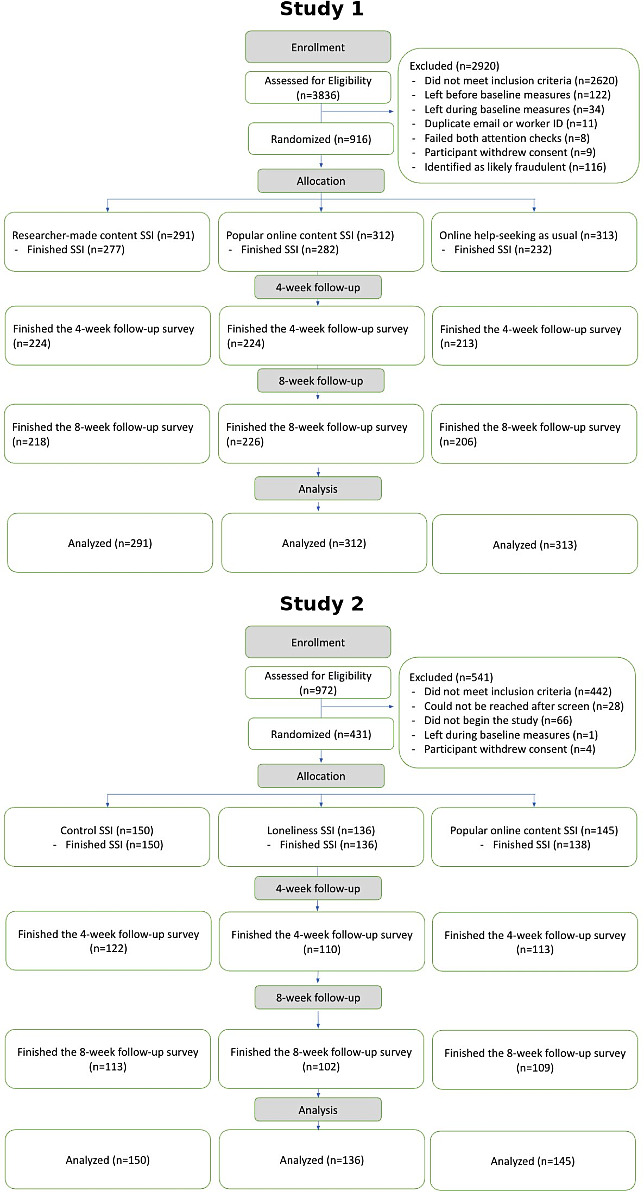
CONSORT flow diagrams for each study showing the number of participants who completed each part of the study.

In study 1, data were collected from April to August 2023. In study 2, data were collected from July to September 2023. Both studies concluded upon reaching (or just after reaching) the preregistered target sample size. A total of 916 participants were randomized in study 1 and 431 in study 2. CONSORT flow diagrams detailing participant randomization and completion at each time point are presented in Figure 2 [19]. In study 1, the median participant age was 32 years; 52.6% (482/916) identified as female, and 63.0% (577/916) identified as White. In study 2, the median age was 36 years; 56.1% (242/431) identified as female, and 68.7% (296/431) identified as White. Baseline demographic characteristics can be accessed through the link provided in Kaveladze [20] for study 1 and in Kaveladze [21] for study 2.

### Feasibility

In study 1, the curated popular online content library’s mean credibility and expected benefit score did not significantly differ from the single-session intervention (Cohen *d*=0.08, 95% CI −0.06 to 0.21; *P*=.88) but was higher than that of unguided online help-seeking (Cohen *d*=0.23, 95% CI 0.10-0.37; *P*=.01). Unguided online help-seeking had a lower mean credibility and expected benefit score than the single-session intervention (Cohen *d*=0.16, 95% CI 0.02-0.29; *P*=.04). Participants were less likely to drop out during the curated popular content library than during unguided online help-seeking (30/312, 9.6% vs 81/313, 25.9%; OR 3.28, 95% CI 2.09-5.17; *P*<.001), although the dropout rate for the curated popular online content library was higher than the single-session intervention (14/291, 4.8% OR 2.10, 95% CI 1.09-4.05; *P*=.03*).*

In study 2, the mean credibility and expected benefit score did not differ between the curated popular online content library and the control (Cohen *d*=0.00, 95% CI −0.14 to 0.15; *P*>.99). The single-session intervention had a higher mean credibility and expected benefit score than both the control (Cohen *d*=0.34, 95% CI 0.20–0.49; *P*=.02) and the curated popular online content library (Cohen *d*=0.32, 95% CI 0.17-0.48; *P*=.02). The dropout rate for the curated popular online content library (7/145, 4.8%) was higher than that of the other 2 conditions (0/136, 0.0% and 0/150, 0.0%; OR 16.30, 95% CI 0.92-288.05; *P*=.001). Because 2 conditions had zero dropout, a continuity correction of 0.5 was applied to calculate the OR. The credibility and expected benefit score across conditions for study 1 can be accessed through the links provided in Kaveladze [22] and in Kaveladze [23] for study 2.

### Impacts on Mental Health Outcomes

Psychological distress in study 1 (*b*=–2.30; Cohen *d*=−0.40, 95% CI −0.33 to −0.46; *P*<.001) and loneliness in study 2 (*b*=–6.06; Cohen *d*=−0.53, 95% CI −0.46 to −0.61; *P*<.001) decreased significantly from baseline to 8-week follow-up. However, changes in primary outcomes over this period did not differ across conditions. In study 1, all between-condition effect sizes were less than 0.15 and all corresponding *P* values were greater than .06. In study 2, all effect sizes were less than 0.12 and all corresponding *P* values were greater than .25. Model outputs and figures for study 1 can be accessed through the links provided in Kaveladze [24] and Kaveladze [25] for study 2. No participants reported harm or unintended effects, although the questionnaires did not include specific items assessing adverse effects.

## Discussion

### Principal Findings

Effectiveness trials of DMHIs delivered online require control conditions that reflect TAU within the online help-seeking context [3]. In secondary analyses of 2 RCTs, the utility of mental health–related popular online content as a TAU control was examined. The findings in this study suggest that curated libraries of popular online content (conceptualized as enhanced TAU) can provide a feasible and ecologically valid control condition in trials of online DMHIs. In study 1, the curated popular online content library outperformed unguided online help-seeking in terms of both dropout as well as the credibility and expected benefit score. In study 2, the credibility and expected benefit score for the curated library did not differ from that of a typical control. However, dropout rates were higher for the curated library than for the single-session intervention in study 1 (30/312, 9.6% vs 14/291, 4.8%) and higher for both the single-session intervention and attention-matched control in study 2 (7/145, 4.8% vs 0/136, 0.0% and 0/150, 0.0%). These findings suggest that the popular online content–based programs may require greater effort than single-session interventions of similar duration and may benefit from further streamlining to facilitate their comparison.

### Strengths and Limitations

Strengths of this study include the preregistered RCT designs and the variety of programs tested. Limitations include the use of paid online worker samples, which may limit generalizability to other populations, reliance on self-reported outcomes, and differences between the samples in the 2 studies. In addition, differential attrition across conditions (particularly in study 1) may have biased the results (ie, if participants who disliked a program were more likely to drop out, their presumably lower CEQ scores would not have been included in that intervention’s mean). Another limitation is that the curated content libraries were culture- and time-specific and were developed through crowdsourcing rather than a more systematic review of popular online content and rigorous evaluation of its effectiveness. Finally, because the studies were underpowered for detecting small effects in brief interventions, small but important differences in efficacy or acceptability across conditions may have been missed.

### Future Directions

One important future direction is the development of improved popular online content controls. Although the curated popular online content conditions in this study functioned as TAU, they were imperfect proxies for typical online help-seeking. Future controls should aim to more closely reflect real-world online help-seeking behaviors, ideally through codesign with individuals who have lived experience of online help-seeking behaviors. One approach may be to integrate the online communities and content-selection algorithms through which help-seekers typically find popular online content. In addition, given the higher attrition observed in the popular online content–based programs, more streamlined interventions (ie, those including only a limited number of content pieces) may also be beneficial. Additional research is needed to minimize the risk of harmful misinformation within popular online content–based programs [4]. Finally, as conversational artificial intelligence chatbots become an increasingly common source of emotional support worldwide, their role as a form of typical online help-seeking should be recognized and accounted for in effectiveness trials.

### Conclusions

To be considered effective in real-world settings, DMHIs delivered online should demonstrate benefit beyond the supportive resources individuals typically access when seeking support online. Popular online content represents a practical and ecologically valid TAU control that may strengthen causal inference in trials of online DMHIs. Future TAU conditions in online help-seeking contexts should aim to align with the effort and attrition profiles of the DMHIs to which they are compared and account for the increasingly prominent role of conversational artificial intelligence in online mental health seeking.

## Supplementary material

10.2196/83707 Checklist 1 CONSORT-eHEALTH Checklist (V 1.6.1).

## Abbreviations

CEQ: Credibility/Expectancy Questionnaire
DMHI: digital mental health intervention
OR: odds ratio
RCT: randomized controlled trial
TAU: treatment-as-usual
